# Clutter Mitigation in Echocardiography Using Sparse Signal Separation

**DOI:** 10.1155/2015/958963

**Published:** 2015-06-24

**Authors:** Javier S. Turek, Michael Elad, Irad Yavneh

**Affiliations:** Department of Computer Science, Israel Institute of Technology (Technion), 3200003 Haifa, Israel

## Abstract

In ultrasound imaging, clutter artifacts degrade images and may cause inaccurate diagnosis. In this paper, we apply a method called Morphological Component Analysis (MCA) for sparse signal separation with the objective of reducing such clutter artifacts. The MCA approach assumes that the two signals in the additive mix have each a sparse representation under some dictionary of atoms (a matrix), and separation is achieved by finding these sparse representations. In our work, an adaptive approach is used for learning the dictionary from the echo data. MCA is compared to Singular Value Filtering (SVF), a Principal Component Analysis- (PCA-) based filtering technique, and to a high-pass Finite Impulse Response (FIR) filter. Each filter is applied to a simulated hypoechoic lesion sequence, as well as experimental cardiac ultrasound data. MCA is demonstrated in both cases to outperform the FIR filter and obtain results comparable to the SVF method in terms of contrast-to-noise ratio (CNR). Furthermore, MCA shows a lower impact on tissue sections while removing the clutter artifacts. In experimental heart data, MCA obtains in our experiments clutter mitigation with an average CNR improvement of 1.33 dB.

## 1. Introduction

In medical ultrasound imaging, a source of artifact called “clutter” is commonly caused by multipath reverberations or off-axis scatterers, and it materializes as a static cloud of echo signals occluding the tissue regions of interest [1, 2]. Clutter artifacts affect the contrast and the readability of images and can induce misleading functional measurements like myocardium strain in cardiac ultrasound and displacement estimation in blood flow imaging. Often, clutter artifacts degrade ultrasound images entailing the use of imaging modalities such as CT or MRI that are more expensive and involve a radiation risk to the patient.

Clutter artifacts from reverberations appear when the acoustic wave bounces back and forth between a reflective structure and the transducer surface. In echocardiography, this is a common phenomenon because the rib cage and the sternum are highly reflective structures in proximity of the path of the acoustic waves to the heart [3]. The energy of the acoustic waves decays with the distance covered and with the number of bounces, such that the effect becomes more significant in the near-field region of the image and less visible in far-field areas. As a consequence, the myocardium is partially occluded by the artifacts, which may lead to wrong cardiac functioning diagnosis through visual inspection or tracking techniques [4, 5]. Methods that overcome the challenges imposed by reverberation echoes include interpolating data from regions of the heart where artifacts are not present [6] or inferring heart motion using probabilistic models for the challenging regions [7]. However, these techniques tend to fail in data from diseased hearts, because abnormal myocardial motion cannot be inferred using statistical assumptions or models. Therefore, a more appropriate methodology may be to separate the clutter from the signal of interest using filtering strategies, allowing motion tracking to be computed in the entire image.

Suggested filtering methods usually involve separation of the tissue and clutter echo signals by linear decomposition. Echo data is transformed to a new coordinate system in which the clutter artifacts and the signal of interest can be separated along different bases or dictionaries. Then, clutter artifacts are suppressed by reducing their respective coefficients while leaving those of the basis of the tissue signal fixed. Existing methods either use a priori orthogonal bases or learn them adaptively from the data. A priori methods use predefined bases that are orthonormal and independent of the data. Commonly used bases are the Discrete Fourier Transform (DFT), which has been used to define FIR or IIR filters for clutter mitigation in blood flow imaging [8, 9], and the wavelet transform for clutter artifact reduction [10, 11]. Also, the short-time Fourier Transform has been used to filter clutter artifacts during beamforming [12].

Although a priori bases are fast to compute, they may produce poor results when clutter and tissue characteristics overlap. Furthermore, physiological differences among patients entail space and time variability for signals characteristics. Adaptive methods have been suggested to overcome these limitations and learn a basis based on the actual data. The predominant method for determining a basis adaptively is the Principal Component Analysis (PCA) that is used to compute a basis based on the covariance characteristics of the data. Usually, adaptive techniques outperform methods that rely on choosing of bases a priori [13–16]. Some methods learn the basis from local areas of the signal [13] without exploiting the whole image information.

In the present paper, a Morphological Component Analysis (MCA) based separation algorithm [17] is introduced to mitigate clutter in ultrasound images while preserving the tissue signal. As described below, the current method learns a nonorthonormal redundant matrix (also called dictionary) from the entire data and decomposes the signal into a linear combination of a few columns (atoms) from the dictionary. Consequently, by separating the dictionary's atoms into clutter and tissue representatives, clutter filtering is achieved by selectively removing clutter atoms. The feasibility of the method is demonstrated with simulated and experimental ultrasound data. Simulation is used to quantify the performance of the method across algorithm parameters and signal motion characteristics. The suggested algorithm is also experimentally demonstrated with echocardiography images, where clutter artifacts are a significant cause of image degradation. Its performance is compared against a high-pass FIR filter and state-of-the-art Singular Value Filtering (SVF) [13].

## 2. Methods

### 2.1. Sparse Representation of a Signal

The sparse representation model [18] assumes that a signal of interest can be decomposed into a linear combination of a few vectors or “atoms” from a given matrix, also called “dictionary.” The atoms that take part in the linear combination are a small subset of the dictionary, and their respective coefficients are called the sparse representation of the signal. This model is used as prior information for signals, where signal reconstruction is performed by first computing the sparse representation of the signal of interest and then by reconstructing the signal from its sparse representation and the dictionary. Selecting the dictionary is an important step in this process and it is usually dependent on the application. The objective is to find an adaptive dictionary that will enable sparse representations of relevant signals as accurately as possible.

The sparse representation principle can be illustrated by considering a signal **t** ∈ *ℂ* ^**n**^, which can be decomposed into a linear combination of atoms:

(1) $$\begin{array}{c} t=Dx=\overset{m}{\underset{i=1}{\sum }}x_{i}d_{i}, \end{array}$$

where vector **x** is the sparse representation of the signal** t**, implying that most entries *x*_*i*_ are zeros, and **d**_*i*_ are the atoms (columns) of the dictionary **D** ∈ *ℂ* ^**n**×**m**^. The sparse vector **x** has ‖**x**‖_0_ = *k* nonzero elements with *k* < *n*. The notation ‖·‖_0_ represents the *ℓ*_0_-norm (usually, the *ℓ*_0_-norm is wrongly known as a quasi- or pseudonorm. The *ℓ*_0_-norm satisfies only two axioms of the norms and thus it should not be considered a norm), that is, the number of nonzero elements in the vector. The set of indices of the nonzero coefficients in **x** is defined as the support *𝒮* and the signal can be decomposed alternatively into **t** = **D**_*𝒮*_ **x**_*𝒮*_, with **D**_*𝒮*_ being the subset *𝒮* of columns from **D** and **x**_*𝒮*_ the reduced vector with only the nonzero elements. When the support of a representation vector is known, the respective coefficients in **x**_*𝒮*_ are computed using the pseudoinverse **D**_*𝒮*_^†^ of the dictionary restricted to the support, **D**_*𝒮*_:

(2) $$\begin{array}{c} t=D_{S}x_{S}⟹x_{S}={\left(\;D_{S}^{*}D_{S}\;\right)}^{-1}D_{S}^{*}t=D_{S}^{†}t. \end{array}$$

The support *𝒮* is unknown in practice and is estimated together with the nonzero coefficient values.

The sparse representation **x** is computed by finding the sparsest vector that yields **t** when multiplied by the given dictionary **D**. This problem can be written as the following optimization task:

(3) min⁡x x0subject  to t=Dx.

In practice, a noisy observation **s** of the signal of interest **t** is obtained. It is often assumed that the noisy signal **s** is contaminated with additive i.i.d. white Gaussian noise **n** ∈ *ℂ* ^**n**^ with noise level *σ*; that is, **s** = **t** + **n**. Problem (3) is then reformulated to yield a solution that is close to the observed signal in the *ℓ*_2_-norm sense:

(4) min⁡x x0subject  to s−Dx22≤ε2,

where *ɛ* is the desired bound on the distance from the observed signal **s** and is usually proportional to the noise standard deviation *σ*. The notation ${\left\|v\right\|}_{2}=\sqrt{\sum_{i=1}^{n}{\left|v_{i}\right|}^{2}}$ represents the *ℓ*_2_-norm of a vector **v**. An alternative to (4) may be formulated where the fidelity data term is minimized and the number of nonzero elements is constrained:

(5) min⁡x s−Dx22subject  to x0≤k0,

where *k*_0_ is the maximum sparsity allowed in each representation. Solving problem (4) or (5) yields an approximate sparse representation $\hat{x}$ and it is used to reconstruct the clean signal by multiplying with **D**; that is, $\hat{t}=D\hat{x}$. The Orthogonal Matching Pursuit (OMP) [19] is a commonly used algorithm designed to approximate solutions to problem (4) or (5), and it is presented in Algorithm 1. The OMP is a greedy pursuit algorithm that increments the support size by one nonzero element at a time. In each iteration, an atom is chosen such that it reduces the residual distance to the observed signal the most. The stopping criterion is given by the constraint of the problem to be solved: the *ℓ*_2_ term error bound for (4) or the number of nonzeros for (5). There are other methods for approximating the solution of (4) or (5); such is the Basis Pursuit method [20] that relaxes the *ℓ*_0_ quasinorm in problem (4) with an *ℓ*_1_-norm (the *ℓ*_1_-norm of a vector **v** is defined as ‖**v**‖_1_ = ∑_*i*=1_^*n*^|**v**_*i*_| and it is well known [18, 20] to give preference to sparse solutions) and solves a convex optimization problem.

In ultrasound imaging, sparse representations have been used widely with interesting results. Zhang et al. [21] used Gabor atoms to denoise Doppler ultrasound blood flow signals. Also, Nieblas et al. [22] used the same dictionary to detect heart pathologies in heart sound signals with high accuracy. Furthermore, Michailovich and Adam [23] separated harmonic components using Gabor frames. Deka and Bora [24] used an adaptive dictionary for despeckling of ultrasound images. Liebgott et al. [25] applied the compressed sensing technique to reconstruct RF ultrasound signals using a dictionary of wave atoms. Also, Shi et al. [26] demonstrated compressed sensing for separating transmitted echoes and improving the resolution in ultrasound flaw detection. Wagner et al. [27] and also Chernyakova and Eldar [28] demonstrated techniques to apply compressed sensing to beamforming achieving a reduction in the sampling rate. Similarly, Zhang et al. [29] proposed an adaptive beamforming approach based on compressed sensing. Zhou et al. [30] developed an asynchronous compressed beamformer that requires low runtime complexity for computing the sparse representations, allowing the authors to use it in portable ultrasound devices. Schiffner et al. [31] used sparse representations with a curvelet dictionary for solving the inverse scattering problem in diagnostic ultrasound images. Richy et al. [32] demonstrated a method for reconstructing the Doppler signal segment by segment in blood flow estimation that is based on compressive sensing using Fourier or wave atom dictionaries. Demirli and Saniie [33, 34] showed how to sparsely decompose ultrasound echo data using envelope and instantaneous phase for identification of signal features and data partitioning. A particular example is the work of Cloutier et al. [35]. In that work, the authors proposed to use the Matching Pursuit approximation algorithm (closely related to OMP) with Gabor atoms as an a priori dictionary to reduce clutter in Doppler blood flow signals. All these works showed performance improvements by assuming sparsity as a signal prior.

The sparse representation clearly changes with selection of the dictionary **D**. There are, as noted above, two ways to select a dictionary, either defined a priori or learned adaptively from the data. Dictionaries such as the Discrete Fourier Transform (DFT) [36] and various types of wavelets such as curvelets [37], countourlets [38], and Gabor wavelets [39], among others, have been suggested as a priori dictionaries for image processing. Many of these options have a fast transformation that allows for a fast computation of a matrix-vector multiplication. However, in terms of quality, these dictionaries may limit the performance of the application [40]. For example, in an application such as ultrasound clutter filtering, using the DFT for source separation may be of limited value because tissue and clutter overlap in the frequency domain. Finding an a priori dictionary for a specific application may not be a trivial task. Alternatively, an adaptive dictionary can be learned from the data and may yield improved results.

A commonly used method to learn a dictionary from data is the K-SVD [41] technique. K-SVD is an iterative method that learns the atoms in a dictionary by fitting the data with the sparsest possible representations. As a consequence, computing an adaptive dictionary with K-SVD requires multiple data samples.

Consider the following example to illustrate how a dictionary is determined with K-SVD. Let **S** ∈ *ℂ* ^**n**×**P**^ be a matrix with *P* data samples ordered so that each sample appears as a column. The number of data samples *P* is usually much bigger than the data dimension *n*, enabling training a dictionary where each atom is used in several representations of samples. The K-SVD method aims to find the best dictionary **D** and the sparse representations **X** ∈ *ℂ* ^**m**×**P**^ (**x**_*i*_ for each sample **s**_*i*_) by solving the optimization problem

(6) min⁡D,X S−DXF2subject  to xi0≤k0,1≤i≤P dj22=1,1≤j≤m,

where *k*_0_ is the maximum sparsity prescribed for each sample representation. To compute a solution to the optimization task in (6), the K-SVD method iterates between two main computational steps: sparse coding and dictionary update. The sparse coding step assumes that the dictionary **D** is fixed and the sparse representations **X** are computed. For instance, this can be done using the OMP algorithm. The dictionary update step modifies each atom one at a time. Hence, the update is computed by isolating the *i*th atom **d**_*i*_ from the others and rewriting the Frobenius norm:

(7) S−DXF2S−∑j≠idjxTj−dixTiF2=Ei−dixTiF2,

where (**x** ^*T*^)_*i*_ stands for the *i*th row of **X**. The terms in parentheses can be considered an error matrix **E**_*i*_ = **S** − ∑_*j*≠*i*_ **d**_*j*_(**x** ^*T*^)_*j*_, where all the elements are fixed and are independent of atom *i*. The optimal solution for **d**_*i*_ and (**x** ^*T*^)_*i*_ that minimizes the functional in (7) is computed by solving a rank-1 approximation of **E**_*i*_ using the Singular Value Decomposition (SVD). In general, the SVD computation yields a dense vector (**x** ^*T*^)_*i*_ which would increase the number of nonzero elements in (**x** ^*T*^)_*i*_ and make use of the updated atom **d**_*i*_ for all the samples. Therefore, the error matrix **E**_*i*_ is restricted to those columns **E**_*i*_^*R*^ where the atom is active, that is, for the nonzeros in (**x** ^*T*^)_*i*_. In this manner, the rank-1 problem is solved by updating only the nonzero elements in (**x** ^*T*^)_*i*_ with the respective columns **E**_*i*_^*R*^. Once all the atoms are updated, the process repeats until some stopping criterion is satisfied. The K-SVD algorithm is presented in Algorithm 2.

### 2.2. Sparse Signal Separation for Clutter Mitigation

A general approach to separate signals using sparse representations is called Morphological Component Analysis (MCA) [17]. In MCA, the mixed signal is decomposed into different morphological components (subdictionaries) and each source is sparse under these subdictionaries. The morphological components can be selected a priori for specific tasks and type of data [42] or can be adaptively learned [17] using some dictionary learning method such as [43, Ch. 15], [44]. Filtering a component is then applied by assigning a weight to each atom that corresponds to that component.

**Figure 1:**
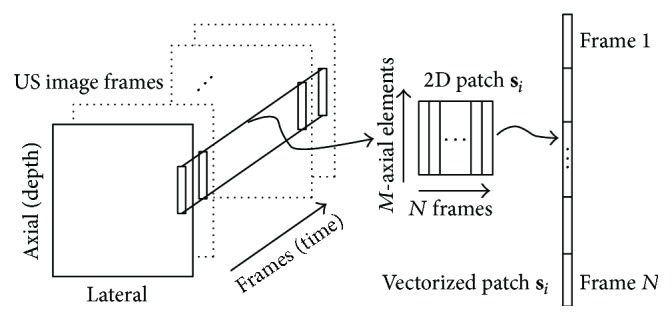
Illustration of how a patch **s**_*i*_ is extracted from a sequence of several frames of complex echo data. The small rectangles in the ultrasound image frames represent *M*-axial elements in each frame which are concatenated to form the patch **s**_*i*_.

While MCA is a general scheme for signal separation, from now on, the discussion will concentrate on clutter artifact mitigation in the context of echocardiography ultrasound imaging. In this application, the observed signals **S** are columned versions of axial-temporal dimensional patches of real-valued raw RF or complex-valued IQ demodulated RF echo data. A signal **s**_*i*_ represents a two-dimensional patch obtained from echo data with *N* consecutive frames as its columns and *M* elements in the axial direction forming its rows (see Figure 1). (The echo data can be taken also as lateral-temporal patches. Clutter quasistatic behavior appears in the temporal dimension, while moving tissue (in any direction) appears as varying elements across the same dimension. This information is captured by such patches as well. Also, data can be taken as a three-dimensional element, by adding consecutive axial lines into every signal **s**_*i*_ and hence including adjacent A-lines in the lateral direction. This also requires us to modify the method for partitioning the atoms in the dictionary into tissue and clutter. In our experiments we found the axial-temporal patches to be slightly more effective.) In this way, every signal **s**_*i*_ contains information of the local motion characteristics in the echo data. However, the number of columns in the patch, *N*, dictates how much of this motion is captured. As clutter behaves quasistatically across frames, a patch with a large number of columns *N* may contain big motion variability making it difficult to remove clutter, whereas a small *N* may not contain enough tissue variability to differentiate it from the clutter artifacts. It should be noted that the amount of motion in the patch depends also on the frame rate of the acquisition. Once the data is separated, the clutter artifacts are removed, the processed signals are converted back from column vectors into two-dimensional patches, and these patches are merged to form the cleaned signal. Then, the process is repeated for the next frame.

Filtering an ultrasound sequence with MCA requires us to define the signal model and assumptions in order to construct the components in the dictionary **D**. The signal model for an observed sequence of echo data **s** is assumed to be a linear superposition of the tissue **t** and clutter **c** subsignals and an additive white noise **n**; that is,

(8) $$\begin{array}{c} s=t+c+n. \end{array}$$

This assumption holds when the signal **s** is an RF or an IQ signal. On the other hand, an envelope-detected signal does not satisfy this model as the absolute value operation for computing the envelope of the signal ruins the linearity assumption.

Additionally, it is assumed that, for any patch, the corresponding **t**_*i*_ and **c**_*i*_ subsignals are sparsely generated by sparse coefficient vectors **x**_*t*_*i*__ and **x**_*c*_*i*__ multiplied by the subdictionaries **D**_*t*_ and **D**_*c*_, respectively. In other words, this assumption means that every patch can be written in the form

(9) $$\begin{array}{c} s_{i}=t_{i}+c_{i}+n_{i}=D_{t}x_{t_{i}}+D_{c}x_{c_{i}}+n_{i}, \end{array}$$

suggesting that clutter is separable from tissue using MCA. The second assumption is that echoes from clutter artifacts are quasistatic, meaning that the subsignal **c**_*i*_ has a quasiconstant pattern in the temporal axis, while the tissue subsignals reflect motion or variability [1].

**Figure 2:**
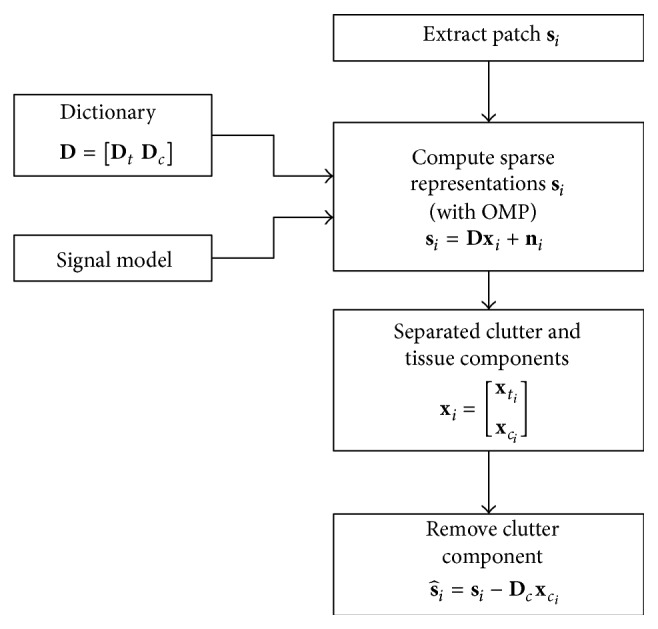
Block diagram of Morphological Component Analysis for clutter reduction for a signal patch **s**_*i*_.

Clutter reduction in a patch **s**_*i*_ is achieved by removing the clutter component **D**_*c*_ **x**_*c*_*i*__ from it; that is,

(10) $$\begin{array}{c} {\hat{s}}_{i}=s_{i}-D_{c}x_{c_{i}}, \end{array}$$

where ${\hat{s}}_{i}$ is the resulting patch with reduced clutter. This requires computing the sparse representations **x**_*t*_*i*__ and **x**_*c*_*i*__ in (9). Note that (9) can be rewritten as follows:

(11) $$\begin{array}{c} s_{i}=\left[\;D_{t}\;|\;D_{c}\;\right]\left[\;\frac{x_{t_{i}}}{x_{c_{i}}}\;\right]+n_{i}=Dx_{i}+n_{i}, \end{array}$$

where **D** is the concatenated dictionary with the tissue and clutter subdictionaries and **x**_*i*_ is the concatenation of the sparse representations of the tissue and clutter signals of the patch. Consequently, solving (4) or (5) with the concatenated dictionary **D** yields the concatenated sparse representation **x**_*i*_. The representations **x**_*t*_*i*__ and **x**_*c*_*i*__ are obtained from **x**_*i*_ relatively to the tissue or clutter atom positions in the concatenated dictionary. In this work, the Orthogonal Matching Pursuit (OMP) [19] algorithm is used to find an approximation to the sparse vector **x**_*i*_. The complete clutter reduction procedure for a patch **s**_*i*_ is illustrated in Figure 2. Additionally, for solving the problem in (4) or (5), the dictionary **D** must be known. An adaptive dictionary **D** allows the method to learn the patient's own physiological characteristics and improve the results. Hence, such a dictionary **D** is learned adaptively from the signal patches {**s**_*i*_}_*i*=1_^*P*^ using the K-SVD algorithm [41].

In order to separate the sparse signals in **x**_*i*_ into the tissue **x**_*t*_*i*__ and the clutter **x**_*c*_*i*__ parts, the division of **D** into the two subdictionaries, **D**_*t*_ and **D**_*c*_, needs to be known. The quality of the separation depends on dividing the dictionary obtained from the K-SVD algorithm into these two disjoint groups. For this purpose, the assumption that clutter artifact echoes are nearly static in time in comparison to the tissue echoes that move is used to differentiate between the atoms in the dictionary (a followup of the current work was recently published in [45], which presents an alternative method to train the dictionaries **D**_*c*_ and **D**_*t*_ separately). As the dictionary **D** is learned from columned versions of 2D (axial-temporal) patches from continuous frames of the measured signal, the learned atoms emulate the behavior of the patches in these dimensions. Therefore, an atom from **D** can be reshaped from a column vector into a 2D (axial-temporal) matrix with the same size of the signal patches. The quasistatic behavior of the clutter artifacts appears in a reshaped atom as a nearly constant pattern across the temporal dimension. In contrast, the atoms containing moving tissue vary in the temporal dimension. (If the tissue moves in the axial direction, the movement is captured in the axial dimension of the atom, hence varying across frames in the temporal dimension. On the other hand, a lateral movement of the tissue is translated into intermittent changes in the temporal dimension of the atom. In both cases, an atom varies across frames and has a nonconstant pattern in the temporal dimension.) An atom can be associated with one of the groups by looking at the rank behavior of its 2D matrix. A low-rank matrix means a nearly constant pattern, while a medium-to-high rank matrix suggests a moving tissue atom. Such a low-rank matrix can be revealed by a high ratio between the first singular value and the sum of all the singular values. Using a predefined cut-off value *β* ∈ (0,1), the atoms with the values of the above ratio greater than or equal to *β* are ascribed to the clutter subdictionary **D**_*c*_. Note that when *β* is close to 1, all the atoms are assigned to the tissue subdictionary **D**_*t*_ and no filtering is expected to happen. Contrarily, when *β* tends to 0, the atoms are assigned to the clutter subdictionary **D**_*c*_ and also tissue is filtered out.

## 3. Results

### 3.1. Experiments with Field II Simulator

A Field II simulation experiment [46] in MATLAB (MathWorks Inc., Natick, MA) of a hypoechoic lesion was performed to evaluate the performance of the MCA method for reducing clutter. The simulated lesion had a diameter of 5 mm and a mean scatterer amplitude ratio of −30 dB between the lesion and the background. Reconstruction of ultrasound images took place within a section of 20 mm × 10 mm where the echo data was collected.

Clutter artifact echoes were simulated from a 0.3 mm × 3.5 mm region of scatterers with reflection amplitudes 20 dB above the lesion. Scatterers for clutter artifacts were simulated separately over the same section and located in the center of the hypoechoic lesion. Scatterers outside the hypoechoic lesion were simulated several times to obtain distinct frames. These scatterers had decorrelation and axial motion across frames. Echo decorrelation between frames was achieved using the Cholesky factorization method in [47]. Clutter scatterers were assigned a small axial motion different from that of the lesion. Axial motion was achieved by oversampling echoes at 400 MHz and then downsampling at 40 MHz starting with the sample that achieves the desired subsample shift. Eventually, the hypoechoic lesion echoes were summed with the clutter artifacts echo data and with electronic noise with noise level *σ* (chosen for SNR of −30 dB) to obtain 19 final frames as supported by the model in (8). The default parameters used for the simulation are presented in Table 1.

The MCA method was compared against two other techniques. The first approach is a Finite Impulse Response (FIR) filter that subtracts the previous frame from the current one [48]. This is a high-pass filter applied to the echo data through time axis. The second is the Singular Value Filtering (SVF) method [13] that applies Principal Component Analysis (PCA) to every axial-temporal dimensional patch and filters clutter by soft-thresholding its normalized singular values. It uses a sigmoidal-like function with a cut-off parameter *τ* and a roll-off parameter *α* that controls the shrinkage operator. Although MCA and SVF methods work with local patches of echo data, the SVF method learns the PCA basis functions from each data patch independently.

**Figure 3:**
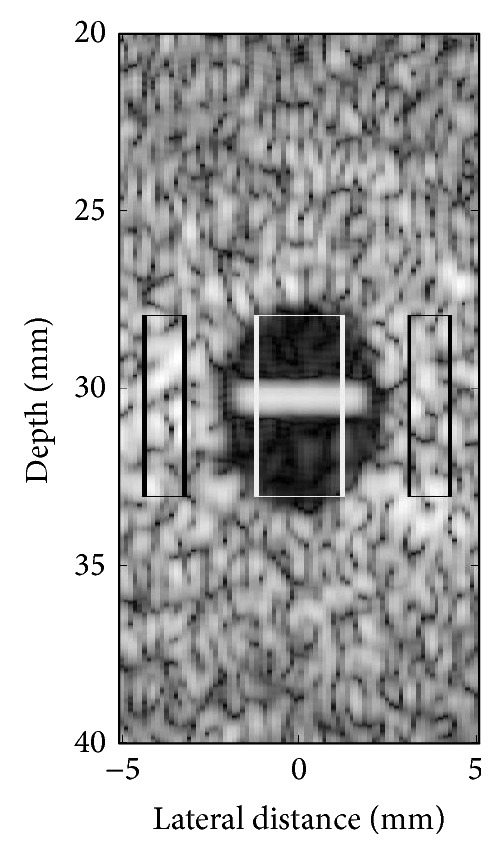
Region of interest used to compute CNR as in (12) for the simulated hypoechoic lesion. The white box corresponds to the ROI inside the lesion with clutter artifacts and the black boxes indicate the ROI outside the lesion.

The resulting performances of the algorithms were measured using contrast-to-noise ratio (CNR). The CNR is defined as

(12) $$\begin{array}{c} \text{CNR}=20\;\log_{10}\left(\;\frac{\left|\mu_{i}-\mu_{o}\right|}{\sigma_{o}}\;\right), \end{array}$$

where *μ*_*i*_ and *μ*_*o*_ are the mean envelope-detected quantities in regions with clutter artifact and without artifacts, respectively, and *σ*_*o*_ is the standard deviation in the clutter-empty region.  Figure 3 shows the regions of interest for computing CNR, the middle box indicating the region with clutter artifacts inside the hypoechoic lesion and the outer boxes indicating the regions outside the lesion. The CNR performance measure may be misleading in some cases. For example, if high values of tissue speckles in the region without artifacts are being reduced, the standard deviation *σ*_*o*_ may decrease faster than the mean *μ*_*o*_ and the ratio |*μ*_*o*_|/*σ*_*o*_ may increase, making the CNR higher. Subsequently, the CNR may exhibit better values than perfect filtered images. Therefore, performance was measured also using peak signal-to-noise ratio (PSNR) in a few particular cases in order to show further differences that exist between the tested methods. PSNR is computed using the following expression:

(13) $$\begin{array}{c} \text{PSNR}=20\;\log_{10}\left(\;\frac{\text{MAX}}{\sqrt{\left(1/n\right){\left\|s-\hat{s}\right\|}_{2}^{2}}}\;\right), \end{array}$$

where MAX is the maximum pixel value in the clean envelope-detected image, **s** ∈ *ℝ* ^**n**^ is the clutter-free envelope-detected signal, and $\hat{s}$ is the reconstructed envelope-detected signal. PSNR measures the distance to the perfect image, penalizing for any difference from it. Thus, it is capable of measuring the remaining clutter as well as the amount of tissue removed. In contrast to CNR, PSNR requires the perfect filtered signal.

**Figure 4:**
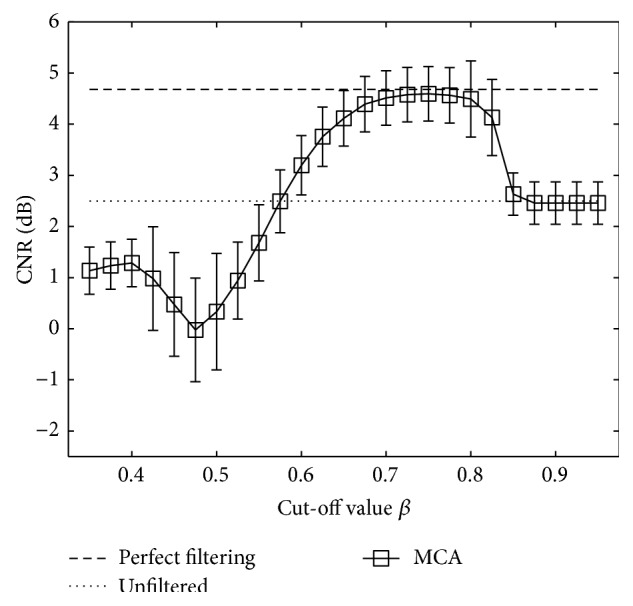
CNR measurements for MCA on complex simulated echo data for several values of the cut-off *β* parameter. Markers represent mean CNR and error bars the standard deviation over 100 simulations.

**Figure 5:**
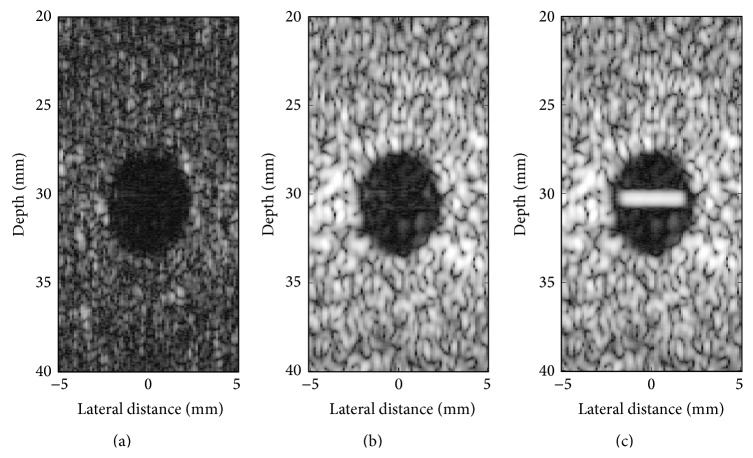
Clutter reduction using MCA on IQ data of simulation images when the cut-off *β* was set to (a) 0.5, (b) 0.75, and (c) 1.

**Figure 6:**
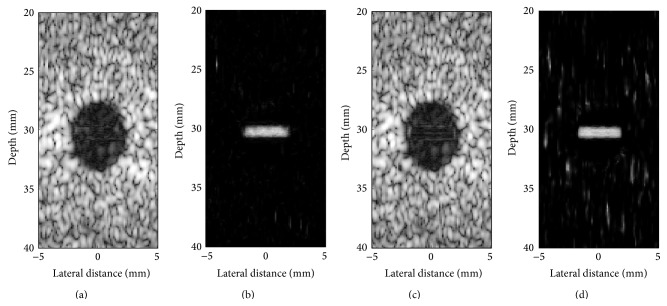
Visual comparison of clutter reduction on IQ data of simulation images obtained by (a) MCA and (c) SVF. The absolute difference between (b) the perfect filtered signal and the MCA reconstruction and (d) the perfect filtered signal and the SVF reconstruction.

**Figure 7:**
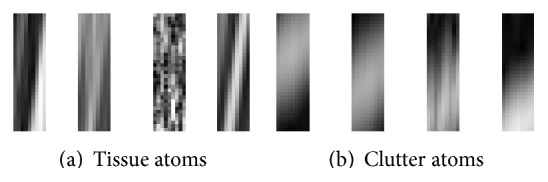
Example of (a) tissue atoms and (b) clutter atoms in the obtained subdictionaries from applying MCA with a cut-off value of *β* = 0.75. The dictionary is learned using the simulated sequence echo data. The magnitude of the atoms is presented. The atoms are shown after being transformed from vectors into 2D patches and being envelope-detected. The horizontal direction refers to the temporal dimension and the vertical direction to the axial dimension.

The influence of the cut-off *β* parameter on separation of tissue from clutter atoms in the dictionary is shown in Figure 4. Mean CNR over 100 trials was computed on complex echo data as a function of the value of the *β* parameter.  Figure 5 presents reconstructed images after MCA for *β* of 0.5, 0.75, and 1. As described in Section 2.2, when *β* is close to one, the data remains unfiltered. In contrast, when *β* tends to zero, most of the data is rejected as clutter and only the noise component remains. Examples of the best reconstructions obtained from the MCA (*β* = 0.75) and the SVF (*τ* = 0.7, *α* = 30) methods are visually compared in Figures 6(a) and 6(c), respectively. The parameters for these methods were selected for the best CNR performance. Additionally, Figures 6(b) and 6(d) present the difference image between the reconstructions obtained by the algorithms and the original image. All simulation images in Figures 3, 5, and 6 are shown on a log compressed linear gray scale mapping to 0 to 30 dB.  Figure 7 shows examples of (a) tissue and (b) clutter atoms in the subdictionaries obtained on one of the simulations with cut-off value *β* of 0.75. The atoms are shown in two-dimensional form with the same size of the patches and after envelope detection, that is, their magnitude. The axial dimension is shown in the vertical direction and the temporal dimension is in the horizontal direction.

**Figure 8:**
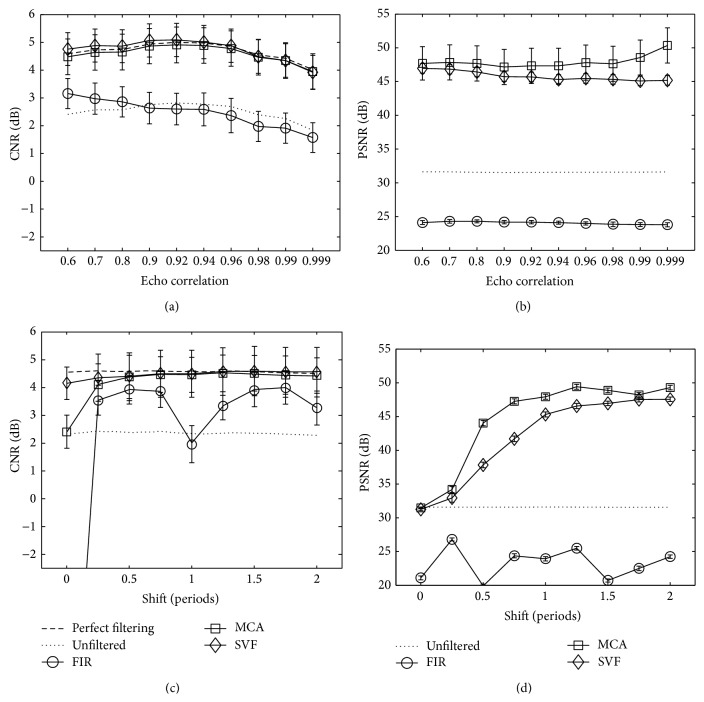
Mean CNR and mean PSNR measurements for MCA, SVF, and FIR methods for varied ((a) and (b)) echo correlation and ((c) and (d)) axial shift of the tissue scatterers. Markers represent the mean CNR and mean PSNR, respectively, and error bars the standard deviation over 100 simulations. The dashed line, labeled “perfect filtering,” represents the mean CNR when the clutter artifacts are not present in the simulated echo data. Likewise, “unfiltered” represents the CNR or PSNR of the measured data with clutter artifacts and when no filtering technique is applied.

**Figure 9:**
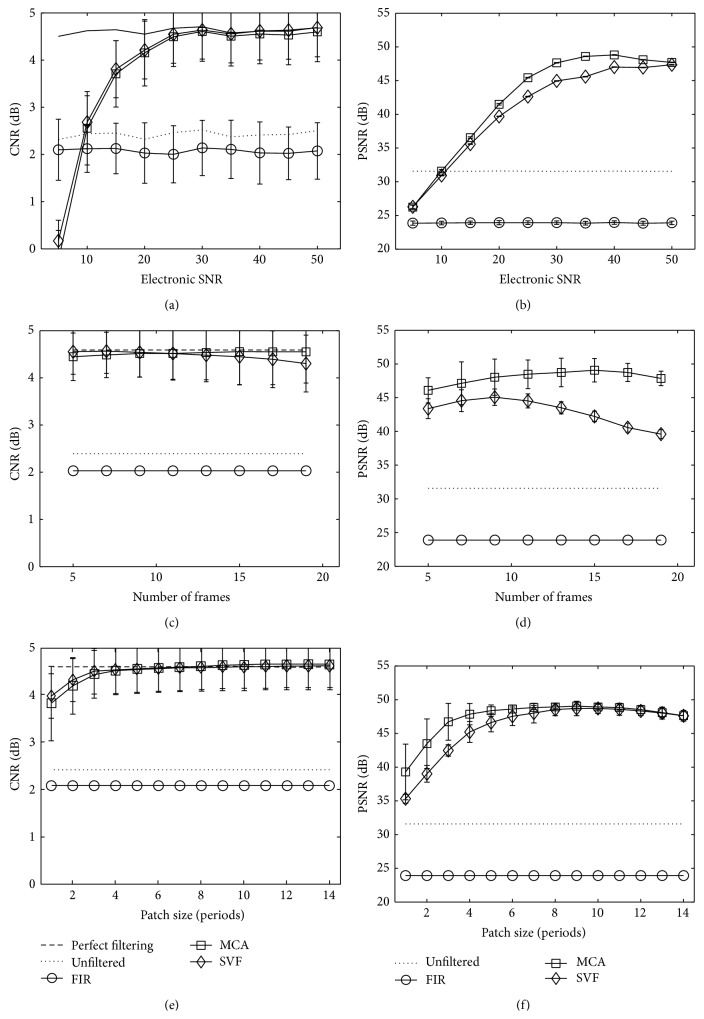
Mean CNR measurements for MCA, SVF, and FIR methods for varied (a) electronic SNR, (b) patch size in temporal dimension, and (c) patch size in axial dimension. Markers represent mean CNR and error bars the standard deviation over 100 simulations. The dashed line, labeled “perfect filtering,” represents the mean CNR when the clutter artifacts are not present in the simulated echo data. Likewise, “unfiltered” represents the CNR or PSNR of the measured data with clutter artifacts and when no filtering technique is applied.

Performance of clutter reduction for the MCA, SVF, and FIR methods on IQ complex echo data measured with CNR and PSNR is presented in Figures 8(a) and 8(b) as a function of tissue echo correlation and Figures 8(c) and 8(d) as a function of tissue axial motion. This simulation shows the influence of different tissue motion and correlation values on the algorithms. The amounts of tissue motion (axial shift) and echo decorrelation serve to simulate the frame rate of an imaging device. The clutter echo correlation and axial shift were held constant at the default simulation values described in Table 1, while the tissue echo correlation and axial shift were modified for the experiment. As a reference, the dashed line labeled “perfect filtering” is included representing the mean CNR for the simulated data without the added clutter artifacts. Additionally, the solid line with label “unfiltered” indicates the mean CNR and PSNR values when no filtering is applied. Markers in the graphs represent the mean CNR or PSNR values, while error bars represent standard deviation over 100 simulations. In Figures 9(a) and 9(b) for CNR and PSNR, respectively, the performance of the algorithms is shown as a function of the electronic signal-to-noise ratio in the simulation. Furthermore, filtering performance for several patch sizes is presented in Figure 9(c) for CNR and Figure 9(d) for PSNR as a function of the size of the temporal dimension and in Figure 9(e) for CNR and Figure 9(f) for PSNR in the axial dimension. Parameters for SVF and MCA were selected for the best performance in each case.

### 3.2. Experiments with Human Heart Images

**Figure 10:**
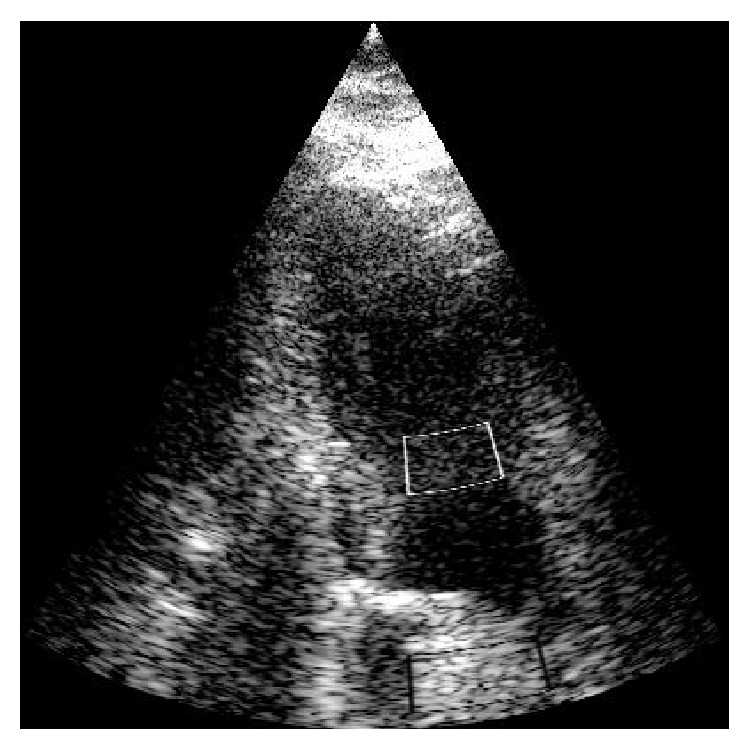
Example of ROI from an apical view of a volunteer heart view for measuring CNR. The upper white box corresponds to the artifact region and the lower black box corresponds to normal tissue.

**Figure 11:**
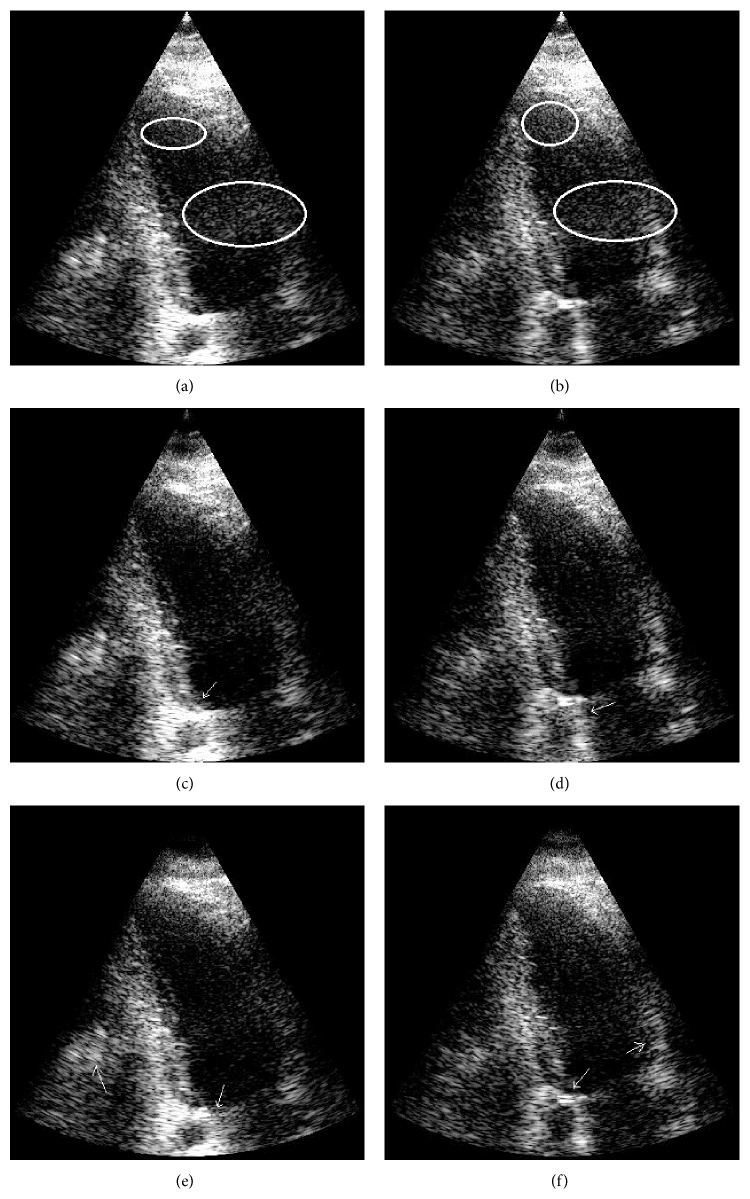
Two examples of an apical view of heart images are illustrated before clutter removal (a, b) and after clutter removal with MCA (c, d) and after applying SVF (e, f). Ellipses indicate regions of clutter artifacts due to multipath reverberations. The arrows point to regions of tissue incorrectly filtered. Images are shown on a log compressed linear gray scale mapping to 0 to 30 dB.

**Figure 12:**
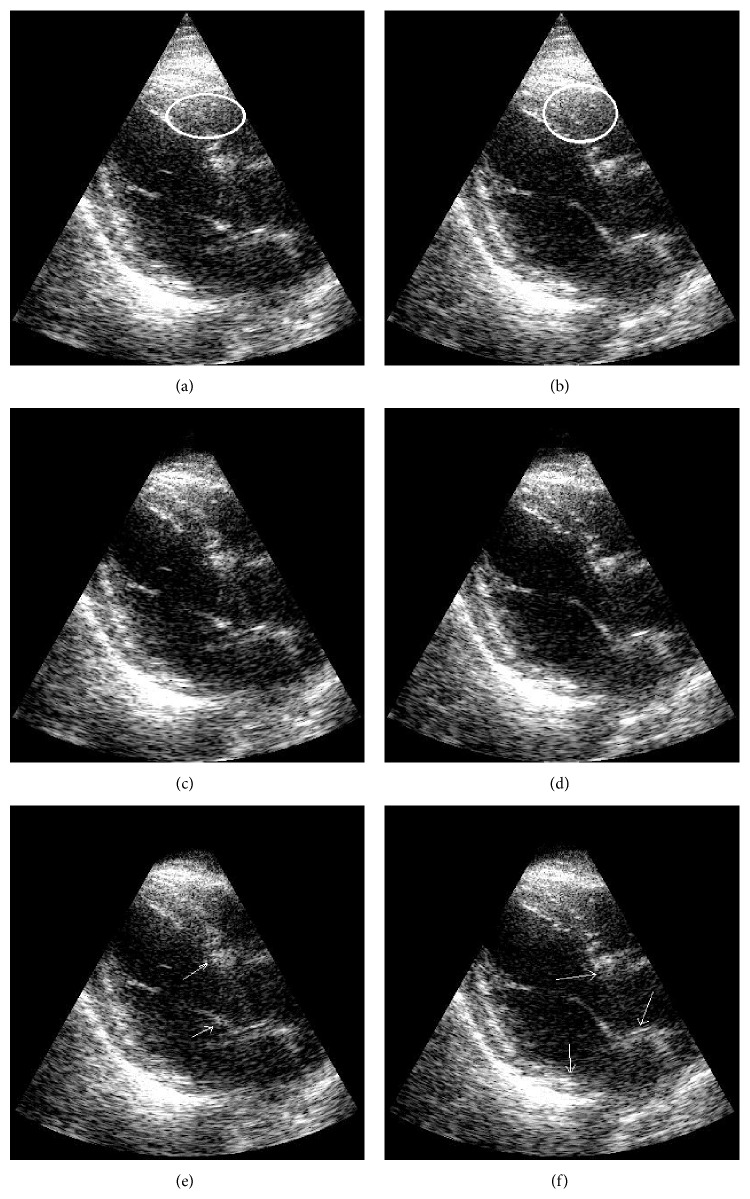
Two examples of an apical view of heart images are illustrated before filtering (a, b) and after clutter removal with MCA (c, d) and after applying SVF (e, f). Ellipses in the unfiltered images indicate regions of clutter artifacts due to multipath reverberations. The arrows point to regions of tissue incorrectly filtered. Images are shown on a log compressed linear gray scale mapping to 0 to 30 dB.

**Figure 13:**
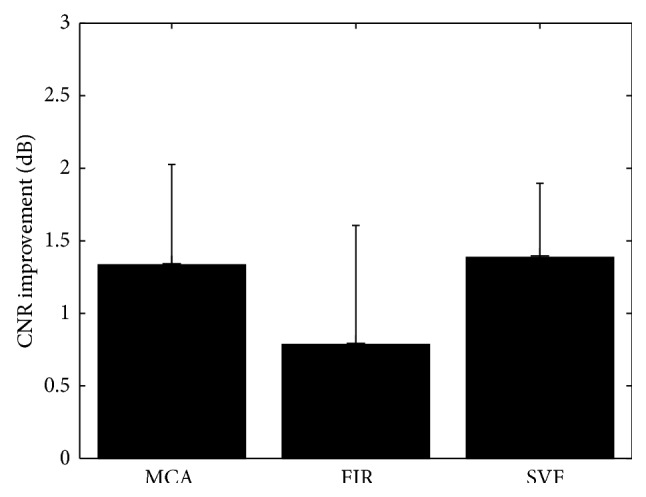
Mean improvement CNR comparison for MCA, SVF, and FIR clutter reduction methods over the unfiltered echo data. Error bars represent standard deviation over eight datasets.

The MCA method was validated experimentally using frames of echo data from apical views of human hearts. The frames were acquired using a Vivid S6 (GE Medical Systems, Israel) ultrasound scanner operating at 3.3 MHz. Clutter artifact was present due to multipath reverberations mainly from the thoracic cage and sternum. Data from a full heart cycle composed of 30 to 40 frames were processed for clutter rejection. The echo sequences were acquired in in-phase and quadrature (IQ) format directly from the Vivid S6 and processed offline using MATLAB (MathWorks Inc., Natick, MA) implementation of the above mentioned three algorithms. Thirteen datasets were acquired from five male volunteers, 30–55 years old. Each dataset included different acquisitions of apical views of the heart to obtain superposed clutter artifacts that were as independent as possible between sets. The resulting performance of the algorithm was measured averaging the CNR over the sequence frames. This metric was used to compare against FIR [48] and SVF [13] methods. The parameter values of the SVF method were set to *τ* = 0.35 and *α* = 25, which were optimized for the best performance. The regions of interest (ROI) for CNR measurements of one example dataset are illustrated in Figure 10. The regions with artifacts used to measure CNR were selected with the advice from an ultrasound technician, and the tissue regions were selected in the far-field region where the tissue is predominant and clutter artifacts are not present. The electrical noise is not known for these datasets. When solving (5), the sparse representations in MCA were allowed a maximum sparsity *k*_0_ of 20% of the patch size. The parameters used to demonstrate the MCA method and compare it to FIR and SVF techniques were a patch size of 15 axial elements and 15 frames in temporal domain with a cut-off *β* at 0.45. Examples of heart images from two datasets are shown in Figures 11 and 12, with the ellipses indicating regions of clutter artifacts. The filtered reconstructions using MCA and SVF are also shown in Figures 11 and 12, respectively. The arrows point to areas where tissue was incorrectly filtered.  Figure 13 compares the mean improvement CNR for the MCA, FIR, and SVF methods over the unfiltered echo data while the error bars represent standard deviation.

## 4. Discussion

The current study demonstrates the potential benefit of the MCA method to remove clutter artifacts that originate from multipath reverberation. The simulation and experimental results quantify the performance of the MCA method compared with a high-pass FIR method [48] and the PCA-based state-of-the-art SVF technique [13]. To our knowledge, MCA or signal separation with a sparsity prior has never been used for clutter removal in B-mode ultrasound. A sparsity prior with a priori dictionary of sinusoids was used to remove clutter in Doppler ultrasound [35].

There are two significant outcomes presented in this paper. The first outcome is the ability to separate the morphological components into clutter and tissue in the learned dictionary for complex echo data. The significance of the temporal dimension in the patches enables the atoms in the dictionary to inherit the motion characteristics of the signals. These motion properties allow us to identify and separate the tissue from the clutter sets of atoms by evaluating their temporal behavior. Because atoms in the dictionary represent the underlying data, they can be used effectively to separate (and filter) tissue and clutter components.  Figure 7 depicts the morphology in the atoms of the obtained dictionary and the motion differences between the clutter atoms and the tissue ones. A separation cut-off parameter *β* was used to separate these morphological components. An example of the effect of this parameter *β* is illustrated in Figure 5. When the value of *β* is too low, the artifacts are filtered but part of the tissue is incorrectly identified as artifact as shown in Figure 5(a). In contrast, when *β* is close to 1, no visible effect is obtained (Figure 5(c)). This effect is also reflected in Figure 4, where CNR is optimized for *β* of 0.75 and performance tends to the CNR value of the unfiltered image as *β* → 1 and to even a lower performance when *β* → 0.

The second outcome is the improved performance using nonorthonormal and redundant bases for filtering. In most applications, adaptive filtering is achieved using PCA-based signal separation [2, 13–15, 49–53], which is an orthonormal basis. In this paper, we have demonstrated that separation performance is improved with a different transformation. Instead of an adaptive orthonormal basis, a redundant dictionary was shown to result in improved image quality as reflected by high CNR values and PSNR values. This redundant dictionary can effectively decompose a signal into a linear combination of a few atoms, allowing the recognition of the tissue and the clutter components in the signal. Thus, filtering only the relevant part of the signal is possible even when clutter is not much stronger than the tissue.

The simulation results show that MCA performs comparably in CNR terms and better in PSNR terms to the state-of-the-art SVF method in all the simulation tests. In Figures 8 and 9, several trends can be observed from the mean CNR and PSNR behavior of MCA as a function of the distinct simulation and algorithm parameters. First, the mean CNR and PSNR performances decrease when the tissue motion characteristics approach those of the clutter artifacts. This is clearly seen in Figures 8(c) and 8(d) when the shift motion of the tissue tends to zero and becomes close to the artifact motion. In such a case, the atoms in the learned dictionary representing tissue and clutter become similar, limiting the ability to correctly identify the atoms partitioning and hence reducing the CNR and PSNR performances. Moreover, the MCA is insensitive to the echo correlation between frames in the sequence, maintaining the same CNR gap with the perfect filtering image (Figure 8(a)). The mean PSNR performance of the MCA shown in Figure 8(b) increases when the echo correlation grows. In contrast, the PSNR performance of SVF remains steady and always below the MCA performance. Results in Figure 9 depict a performance decrease with low electronic SNR and with small patch sizes, both in axial and time dimensions. Figures 9(a) and 9(b) show that MCA and SVF have a similar reaction to low electronic SNR, with MCA having a better mean PSNR performance for middle values. In Figures 9(e) and 9(f), mean CNR and PSNR performances remain stable when the patch size in the axial dimension is at least 3 periods long (24 pixels). This occurs because local statistics are well described when a patch is large enough to include sufficient information. However, the mean PSNR performance depicts better results for MCA for small patches. When it comes to the influence of the number of frames in the patch size in Figure 9(c), the behavior of the MCA method departs from that of the SVF. The MCA method is more robust to longer patches in time because the sparsity prior assumed for the clutter signal helps separate the artifact parts that may vary slightly in time in these longer patches. On the other hand, MCA performance decreased when the size was reduced, because of the tendency to misclassify atoms when there is not enough tissue motion information. Figure 9(d) shows this behavior better and also reveals that MCA obtains PSNR values higher than those of SVF.

In Figures 8(a) and 8(c), the SVF method obtained mean CNR values that were better than the perfect filtered sequence. Such behavior can be explained by the fact that tissue is being removed, reducing the mean tissue *μ*_*o*_ values and their standard deviation *σ*_*o*_. In particular, when “peaks” or high values in tissue are reduced, the standard deviation decreases faster than the mean, increasing the ratio |*μ*_*o*_|/*σ*_*o*_ in formula (12) and thus increasing the CNR performance to higher levels than the perfect filtering. This effect may lead to misleading conclusions because in these circumstances CNR may not be a reliable indicator of contrast improvement. Such a misleading example is shown in Figure 8(d), where the PSNR performance of MCA and SVF for low axial shift is quite the same as the unfiltered image, while in Figure 8(c) SVF achieved higher CNR values than MCA (and the unfiltered data). Another example is presented in Figure 6 that visually compares the reconstructed images using MCA and SVF and the difference image of their respective reconstruction with the perfect filtered image. While the amount of artifacts removed is similar, the amount of tissue removed is higher. The PSNR measure was introduced to overcome the limitations of the CNR formula. PSNR measures the distance to the perfect filtering image, penalizing for removed tissue and for unfiltered clutter artifacts. Therefore, similar CNR values with different PSNR values suggest good clutter filtering with tissue removed for the lower PSNR result. Figures 8(b), 8(d), 9(b), 9(d), and 9(f) illustrate the experiments using the PSNR measure to show to what extent each method preserved the tissue in the overall image. In these figures, it can be seen that MCA preserved the tissue better, while both algorithms removed clutter effectively.

Unfortunately, PSNR cannot be used in real experimental data because the perfect filtered images are needed to compute it. Indeed, the CNR measure suffers from this problem and it is far from ideal to measure contrast improvements for ultrasound medical images. An alternative expression to measure contrast improvements should be developed taking into account the removed tissue and the removed clutter. How to define such a measure is an important question but it is beyond the scope of this paper.

The simulations used in this paper to obtain Figures 8 and 9 have significant limitations. The simulations do not necessarily model the physics of clutter artifacts from multipath reverberations but are used to assess the signal model given as the superposition of clutter, tissue, and noise components. Furthermore, the simulations were not intended to model the human physiology and clutter was placed at the center of the lesion for convenience. Therefore, the results of the simulations may not directly translate to human cardiac imaging. The main value of the simulation study is meant to illustrate the ability of MCA to separate clutter from the tissue component over a wide range of conditions and algorithm parameters.

**Figure 14:**
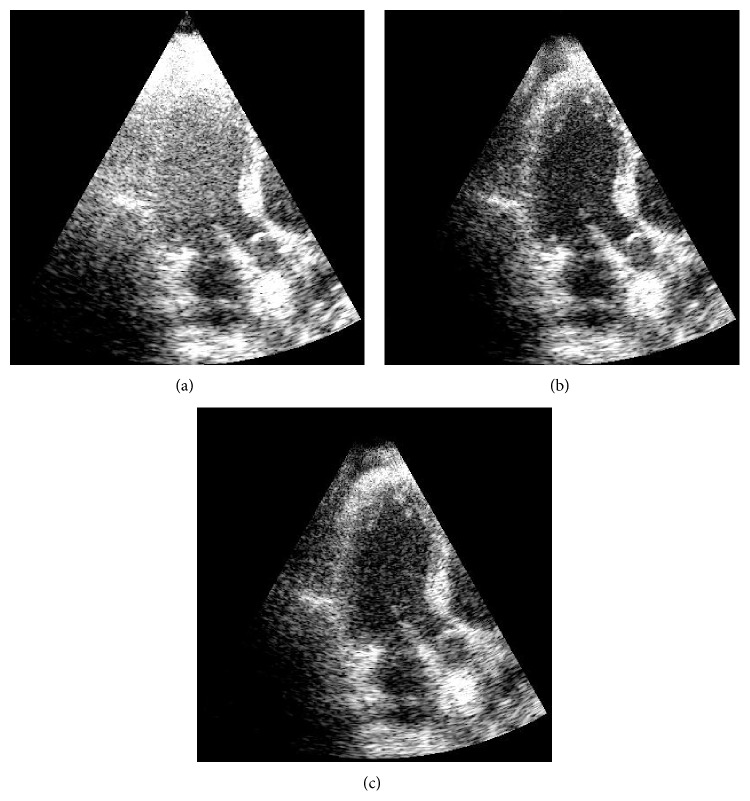
A heavily cluttered apical view of heart images illustrated before filtering (a) and after clutter removal with MCA (b) and after applying SVF (c). Images are shown on a log compressed linear gray scale mapping to 0 to 30 dB.

The MCA technique was demonstrated in experimental human heart data with superior clutter mitigation results. In these datasets, clutter appeared mainly because of multipath reverberations from the thoracic cage.  Figure 13 shows that the MCA obtained an average CNR gain of 1.33 dB, while SVF obtained 1.38 dB and FIR only 0.78 dB. The average performance of MCA in this measure was slightly lower but still comparable to that of SVF. However, when it comes to visual comparison in Figures 11 and 12, the MCA method is seen to remove the same amounts of clutter as SVF, but less tissue than SVF. In all datasets, both MCA and SVF greatly reduced clutter artifacts independent of the clutter place in the image. In particular, better mitigation performance was obtained when the artifacts appear in the blood pool than over tissue, due to an easier recognition of the artifacts' representations. In areas where the clutter occluded the myocardium, the methods managed to reduce the artifacts without removing most or all of the tissue (see Figure 11 as an example). As an example, Figure 14 presents a frame of an additional sequence containing artifacts in the myocardium area and how the MCA (Figure 14(b)) and SVF (Figure 14(c)) methods reveal the myocardium pretty well. However, when the probe is slightly moved the method reduces its filtering performance. The reason is that clutter that moves within a few frames is confused with tissue, and OMP tends to select atoms from the tissue dictionary instead. The same undesired effect happens when applying SVF because such a patch has a higher rank due to the clutter motion. Nevertheless, this effect typically degrades the performance for a few frames, until the probe (and clutter) stops moving. An opposite effect may happen in the frames respective to diastole where the myocardium relaxes and remains quasistatic for a few frames. In this case both MCA and SVF may remove tissue. Consequently, this effect may be scaled down by taking bigger patches in the temporal domain in order to maintain information on the tissue motion before and after the relaxation period, but with performance reduction on the clutter reduction.

One of the limitations of the SVF method is that it needs to compute a singular value decomposition for every element in the echo data. On the other hand, MCA computes a singular value decomposition for every atom in the dictionary **D** one time for the entire sequence, which is much cheaper. However, the computational cost of MCA is dominated by sparse coding of the signals. In this case, the MCA method cost corresponds to that of the OMP method (for learning and filtering) [54]. Nevertheless, the costs can be reduced if the OMP algorithm used to solve (4) or (5) is changed by a faster approximation approach such as solving the Basis Pursuit [20] with fast convex optimization techniques. Computational runtime can be further reduced using parallel implementations, or techniques that increase speed with a small performance reduction such as a smaller patch overlapping.

It is worth mentioning that algorithm parameters for both MCA and SVF were chosen differently for the experimental data than in the simulations. Because of the differences in the characteristics in the type of data, it should be expected that such a difference exists between the experiments. One of the characteristics of the B-mode ultrasound imaging is the frame rate of the acquisition of the echo data. As the patches are taken in the time dimension, frame rate clearly affects the performance of MCA. When echo signals are acquired with a high frame rate the performance of MCA is expected to decrease because the echo correlation between frames increases and the tissue has a lower axial motion, similar to that of the clutter artifacts. In ultrafast plane wave imaging, for example, such difficulties are expected and thus additional treatment, like skipping frames, is needed for this ultrasound imaging mode. The effects of frame rate are demonstrated in the simulation results in Figure 8, where CNR and PSNR performance are quantified as a function of echo correlation and axial shift. Simulation results discussed above show that the MCA performance was stable for a wide range of these parameters and was significantly affected for small axial shift differences between the tissue and the clutter artifacts. The MCA performance deteriorated when the motion features of tissue and clutter signals became similar because of the difficulties of MCA to separate the atoms in the dictionary into the respective sets. In other words, the frame rate has a predominant influence on the optimal cut-off (*β*) value. As frame rate decreases, or heart rate increases, the axial motion difference grows and it is captured better in the dictionary atoms. Therefore, lower *β* values achieve better performance as separation becomes easier. The results in this paper show good simulation and heart dataset performances with fixed cut-off *β* values. Although the *β* value could be adaptively selected to further improve performance in every dataset separately, this was not needed to obtain good performance.

The current MCA algorithm can be extended to remove clutter in Doppler ultrasound methodologies. For example, in the blood flow application, the signal is usually assumed to be a linear superposition of a blood signal, a clutter artifact, and noise. Every echo element is measured in the temporal dimension and thus the signal or one-dimensional patches of it (as suggested in [14]) can be used to learn a dictionary. Based on the low spectrum characteristics and the intensity of the clutter and blood flow signals in Doppler [55], an approach can be suggested for detecting and partitioning a dictionary into clutter and blood flow atoms. Extending MCA to this application is being left for future work.

The appropriate separation using MCA is based on the assumption that each signal has a sparse representation and a dictionary (or “morphological component”) that describes its nature. This further allows for the recognition of the clutter artifacts and tissue atoms in the dictionary based on the differences in their motion characteristics. When clutter artifacts result from multipath reverberations because of the ribs and the sternum, their exhibited motion is lower than cardiac tissue. This observation is the basis for the recognition of each group of atoms. However, the MCA algorithm as presented here is likely to be ineffective in some particular cases. In pathological cases like cardiomyopathy, in which portions of the myocardium can appear to be almost static, there is no possibility to differentiate between the tissue and the clutter artifacts. In such a case, some additional assumption on either the clutter or the tissue is required in order to detect the signal components correctly. Also, clutter may appear as an effect from the cardiac tissue or other structures in similar ultrasound applications, where the motion characteristics are very similar to those of the desirable structures. For example, the rapid movement of the heart valve leaflets may cause transient clutter to appear in Color Doppler with similar properties to those of the blood flow. Such an effect appears as a short temporal band occupying the full velocity range in the Doppler spectrogram. Applying MCA for removing transient clutter can be done by detecting this effect by analyzing the sparse representation coefficients in **x**, or even by assuming a new signal component that represents this undesirable effect.

## 5. Conclusions

The Morphological Component Analysis (MCA) method for signal separation has been presented for clutter mitigation in medical ultrasound. The MCA technique assumes a sparsity prior on the superposed signals. This assumption, together with a powerful dictionary learning method, such as K-SVD, allows us to translate the motion characteristics of the IQ complex echo data into the dictionary atoms. This adaptive dictionary represents an overcomplete set of directions, rather than a unique orthonormal basis as in previous works. Furthermore, it allows for a good representation of the data with its underlying statistics and for high performance filtering of the clutter artifacts. The low-rank test with the cut-off value was demonstrated to be a good separation measure for deciding whether the atoms should belong to the tissue set or to the clutter set. The MCA technique was shown to mitigate clutter artifacts in simulations of a hypoechoic lesion with mean CNR values close to the perfect filtering in a wide range of image parameters. Also, it was shown to outperform a high-pass FIR filter and to obtain results comparable to these of the state-of-the-art SVF method both on simulated and experimental data. The CNR measure was shown to be misleading in some cases, and PSNR was included to show that MCA removes less tissue than SVF. In human heart data, it was shown that a 1.33 dB gain can be obtained using MCA, comparable to that of SVF, but with improved visual results in tissue areas. In conclusion, the MCA technique is a signal separation method that allows for clutter filtering using a sparsity prior with a general and redundant basis and atoms that can be adaptively learnt from the echo data. It may be used in other medical imaging applications, given that we have shown this method to yield improved mitigation of clutter artifacts in ultrasound imaging.

## Figures and Tables

**Algorithm 1:**
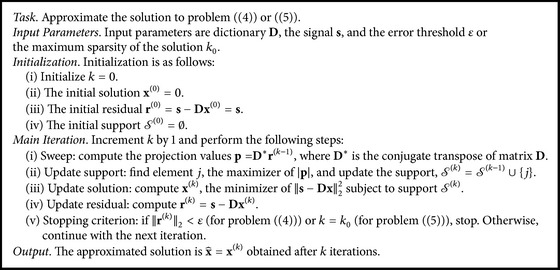
Orthogonal Matching Pursuit.

**Algorithm 2:**
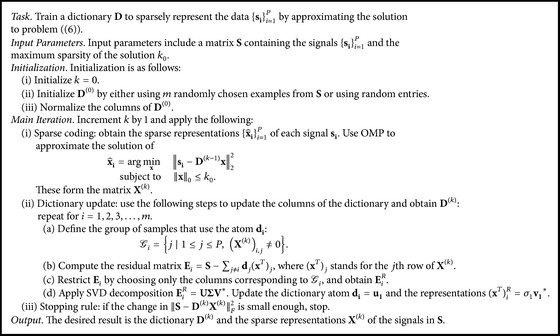
K-SVD.

**Table 1:** Default Field II simulation parameters.

Simulation parameter	Default value
Center frequency	5 MHz
Sampling frequency	40 MHz
Fractional bandwidth	50%
Tissue echo correlation	0.98
Clutter echo correlation	1.0
Tissue displacement	1 period per frame (8 pixels)
Clutter displacement	1/8 periods per frame (1 pixel)
MCA time length (*N*)	9 frames
MCA axial length (*M*)	4 periods (32 pixels)
MCA error threshold	$2.3\sigma \sqrt{2NM}$
MCA dictionary redundancy	2 : 1
MCA patch samples	84640 patches
